# Unraveling the maternal and paternal origins of allotetraploid *Vigna reflexo-pilosa*

**DOI:** 10.1038/s41598-023-49908-2

**Published:** 2023-12-22

**Authors:** Jayern Lee, Yang Jae Kang, Halim Park, Sangrea Shim, Jungmin Ha, Taeyoung Lee, Moon Young Kim, Suk-Ha Lee

**Affiliations:** 1 Department of Agriculture, Forestry and Bioresources and Research Institute of Agriculture and Life Sciences, Seoul National University, Seoul, Republic of Korea; 2 Division of Bio & Medical Bigdata Department (BK4 Program), Gyeongsang National University, Jinju, Republic of Korea; 3 Department of Forest Resources, College of Forest and Environmental Sciences, Kangwon National University, Chuncheon, Republic of Korea; 4 Division of Life Science Department at, Gyeongsang National University, Jinju, Republic of Korea; 5 Department of Plant Science, Gangneung-Wonju National University, Gangneung, Republic of Korea; 6 Macrogen, Seoul, Republic of Korea; 7 Plant Genomics and Breeding Institute, Seoul National University, Seoul, Republic of Korea

**Keywords:** Computational biology and bioinformatics, Evolution, Genetics, Plant sciences

## Abstract

The genomic structures of *Vigna hirtella* Ridl. and *Vigna trinervia* (B.Heyne ex Wight & Arn.) Tateishi & Maxted, key ancestral species of the allotetraploid *Vigna reflexo-pilosa* var. *glabra* (Roxb.) N.Tomooka & Maxted, remain poorly understood. This study presents a comprehensive genomic comparison of these species to deepen our knowledge of their evolutionary trajectories. By comparing the genomic profiles of *V. hirtella* and *V. trinervia* with those of *V. reflexo-pilosa*, we investigate the complex genomic mechanisms underlying allopolyploid evolution within the genus *Vigna*. Comparison of the chloroplast genome revealed that *V. trinervia* is closely related to *V. reflexo-pilosa*. De novo assembly of the whole genome, followed by synteny analysis and Ks value calculations, confirms that *V. trinervia* is closely related to the A genome of *V. reflexo-pilosa*, and *V. hirtella* to its B genome. Furthermore, the comparative analyses reveal that *V. reflexo-pilosa* retains residual signatures of a previous polyploidization event, particularly evident in higher gene family copy numbers. Our research provides genomic evidence for polyploidization within the genus *Vigna* and identifies potential donor species of allotetraploid species using de novo assembly techniques. Given the Southeast Asian distribution of both *V. hirtella* and *V. trinervia*, natural hybridization between these species, with *V. trinervia* as the maternal ancestor and *V. hirtella* as the paternal donor, seems plausible.

## Introduction

Polyploidy is a remarkable biological phenomenon characterized by the presence of more than two sets of chromosomes in an organism^1^. Among the different types of polyploidy, allotetraploidy occurs when two different genomes combine as a result of hybridization between different species or varieties. The effects of allotetraploidy on plants include changes in gene expression, increased size and growth rate, and altered reproductive behavior^2,3^. As a result, it has been the focus of extensive research in the plant sciences^4,5^.

Soybean (*Glycine max*), an important legume crop, exhibits allotetraploidy and has been extensively studied due to its agricultural importance^6^. The availability of a reference genome for soybean^7^ has facilitated these studies; however, understanding the genetic components provided by the ancestral species remains elusive^8^. As a result, the distribution and evolutionary history of donor genomic components in allotetraploid soybean are not fully understood.

Following polyploidization, diploidization and fractionation mechanisms are expected to mitigate the potentially deleterious effects of increased gene dosage on plant adaptability^9^. Diploidization involves halving the chromosomal complement of the polyploid genome, resulting in a diploid-like genome capable of restoring regular meiotic processes and sexual reproduction^10^. Fractionation, on the other hand, refers to the selective loss of redundant or nonessential genes following polyploidization^11^. This process reduces gene dosage and mitigates potential genetic imbalances caused by gene duplication^11^. Through the rationalization of their genetic makeup, plants have the capacity to shape their own genome architecture, influencing the emergence of novel gene functionalities, regulatory networks, and phenotypic traits during evolution^12^. This type of large scaled chromosome rearrangement and rebuilding has been documented in previous studies involving *Brassica napus*^13,14^, *Tragopogon* allopolyploids^15^, and *Pyrus bretschneideri*^16^.

To gain valuable insights into the gene-level consequences of diploidization and fractionation processes in the context of known donor and allotetraploid species, it is imperative to study genetic interactions in species with known genetic backgrounds.

The *Vigna*, a genus within the legume family, encompasses over 100 plant species, with agronomic importance attributed to certain key species such as cowpea, mungbean, azuki bean, bambara groundnut, moth bean, and rice bean. Cultivated primarily in warm temperate and tropical regions worldwide, these crops are renowned for their grains rich in easily digestible proteins. Moreover, they serve diverse agricultural purposes, including as forage, green manure, and cover crops. The crops' short life cycle renders them suitable for catch cropping, intercropping, mixed cropping, or relay cropping. Despite the development of improved cultivars, the full yield potential of various *Vigna* crops is hindered by persistent challenges posed by biotic and abiotic stresses^17^.

The *Vigna reflexo-pilosa* var. *glabra* (Roxb.) N.Tomooka & Maxted contributes to the study of genomic consequences of polyploidization within the genus *Vigna* due to its distinctive characteristics within the genus, known for its polyploidy nature, setting it apart from the predominantly diploid composition of others. *V. reflexo-pilosa* is an allotetraploid formed through hybridization from two genome donors, exhibiting distinct differences in flower, leaf, seed size, and other characteristics compared to the species in genus *Vigna*. Additionally, it demonstrates strong resistance to several insect pests and diseases, including bruchids, bean fly, powdery mildew, and cucumber mosaic virus^18,19^. While not widely distributed as a food crop, there are cases where research has been conducted to introduce genes of *V. reflexo-pilosa* into mungbean^20^. Also, understanding how allotetraploidization has shaped the genetic diversity and adaptive capacity of *Vigna* species has important implications for crop improvement. The diversity within this genus provides an opportunity to study the genetic outcomes associated with allotetraploidization. Some species within the genus, such as *Vigna hirtella* Ridl. and *Vigna trinervia* (B.Heyne ex Wight & Arn.) Tateishi & Maxted, have been proposed as potential ancestors of *V. reflexo-pilosa*^21,22^, highlighting the importance of *Vigna* for studying these evolutionary dynamics. A phylogenetic analysis based on simple sequence repeats (SSRs) showed that specific taxa of *V. hirtella* and *V. trinervia* contributed their genomic components to *V. reflexo-pilosa*^22^. Furthermore, morphological variation, with *V. reflexo-pilosa* being nearly twice the size of *V. hirtella* and *V. trinervia*, suggests that polyploidization may play a role^23^.

We aimed to identify the potential genome donors to *V. reflexo-pilosa*. Using chloroplast genome sequence from RNA-seq of *Vigna* species and high quality reference nuclear genome sequence were compared to elucidate gene-level evidence for allopolyploidization within the genus *Vigna* Through these efforts, we expect to gain a deeper understanding of the complexities involved in polyploidization and genome evolution.

## Results

### Consensus sequences of the chloroplast genomes of 23 *Vigna* accessions

**Figure 1:**
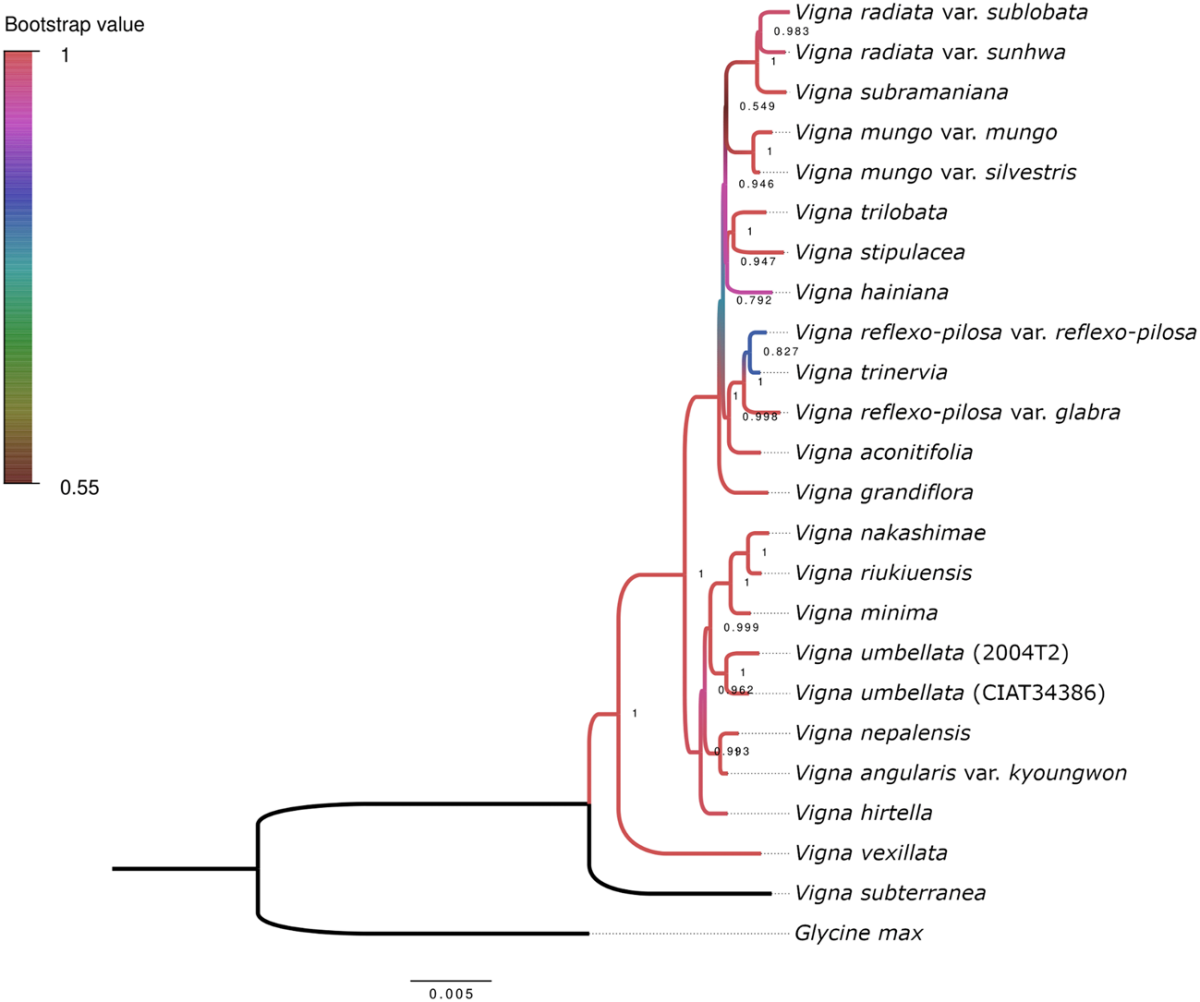
Phylogenetic reconstruction based on single copy chloroplast genes, using protein inference derived from transcriptomic data. This tree elucidates the evolutionary relationships among the 23 *Vigna* accessions and provides insights into their possible genetic neighborhoods.

**Figure 2:**
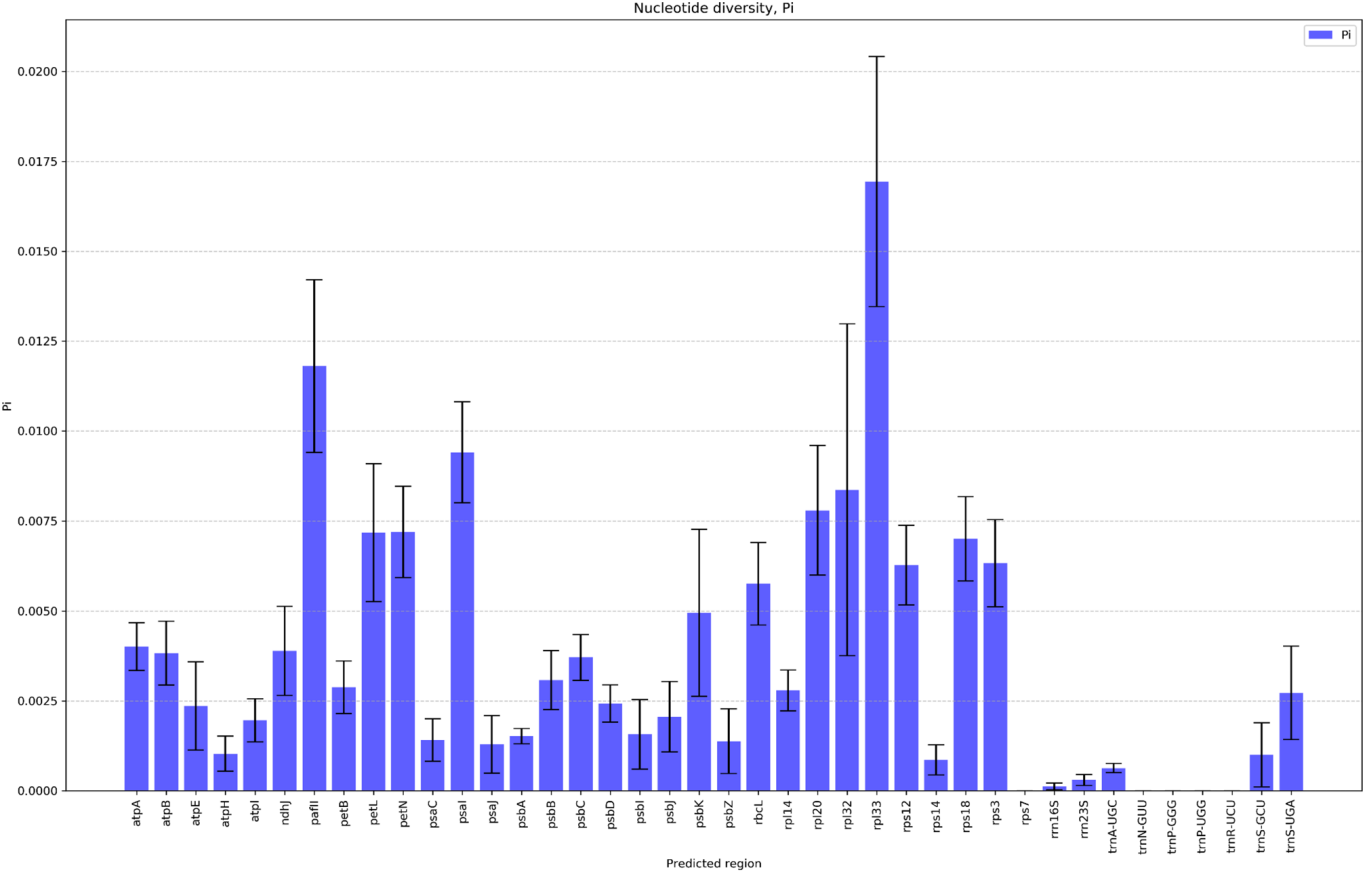
Nucleotide diversity (Pi) values with standard deviation for 40 predicted genes among 23 *Vigna* accessions. The diversity level among species for rpl33 appears to be the highest, while rps7, trnN-GUU, trnP-GGG, trnP-UGG, and trnR-UCU have been identified as well conserved within the genus *Vigna*.

The newly constructed phylogenetic tree, utilizing consensus sequences from chloroplasts, exhibited a correspondence with major clades and consistent interorganism relationships^21,24^. This alignment provides robust evidence supporting the accuracy and reliability of RNA-seq data in capturing evolutionary signals (Fig. 1). Approximately 140 genes were predicted in each consensus sequence representing different accessions. Among these, 40 genes were found to be covered by the RNA-seq reads. When gene-level nucleotide diversity (Pi) values were calculated for these common regions, rpl33 showed the highest value of 0.01694 (Fig. 2). In addition, we constructed a phylogenetic tree using the given sequences. The results of comparing this tree to the phylogeny result of consensus sequences revealed a Normalized Robinson-Foulds (nRF) score of 0.20 and a Robinson-Foulds (RF) score of 8.0. The Maximum Robinson-Foulds (maxRF) score was found to be 40.0. Additionally, both the source tree (src-br +) and the reference tree (ref-br +) demonstrated branch support values of 0.90.

*V. trinervia*, previously identified as the ancestor of *V. reflexo-pilosa*, was confirmed to be very closely related. In addition, *V. hirtella*, newly analyzed in this study, was found to belong to the *Angulares* group^24^, a section of Asian *Vigna* that encompasses *Vigna angularis*, *Vigna riukiensis*, *Vigna minima*, *Vigna umbellata*, and *Vigna nepalensis*. However, it showed a clear genetic distance from the other five species.

**Figure 3:**
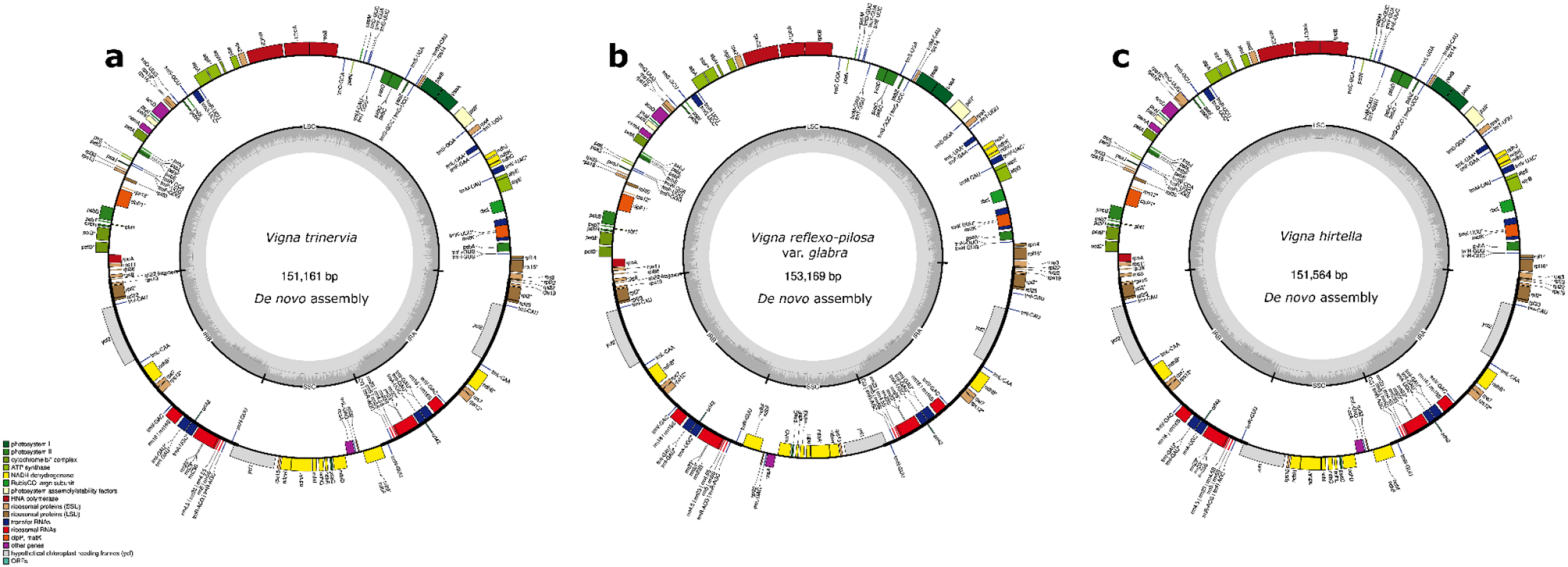
De novo assembly and gene prediction results of the chloroplast genomes of (**a**) *V. trinervia*, (**b**) *V. reflexo-pilosa,* and (**c**) *V. hirtella*. Genes on the inner circle are transcribed in a clockwise direction, while those on the outer circle are transcribed counterclockwise. The darker gray shade on the inner circle indicates the presence of GC content, while the lighter gray shade represents AT content.

The chloroplast genomes were assembled with sizes of 153,169 bp for *V. reflexo-pilosa*, 151,161 bp for *V. trinervia*, and 151,564 bp for *V. hirtella* (Fig. 3). Consensus sequences, generated by RNA-seq measured 151,185 bp, 151,151 bp, and 151,211 bp, respectively. Although some variations were observed, such as the presence of psbM in the *V. hirtella *de novo assembly result, which was not confirmed in the consensus sequence, overall the sequence of key components was well matched at the genetic level. Using BLAST alignment against the chloroplast genome de novo assembly results of *V. trinervia*, *V. hirtella*, and *V. reflexo-pilosa*, 38 genes derived from the *V. trinervia* chloroplast consensus sequence, 34 genes from the *V. hirtella* chloroplast consensus sequence, and 37 genes from the *V. reflexo-pilosa* chloroplast consensus sequence showed the highest sequence similarity to their respective genomes among the 23 accessions used for Pi calculations (Supplementary Table S1).

### Whole genome de novo assembly and annotation

**Figure 4:**
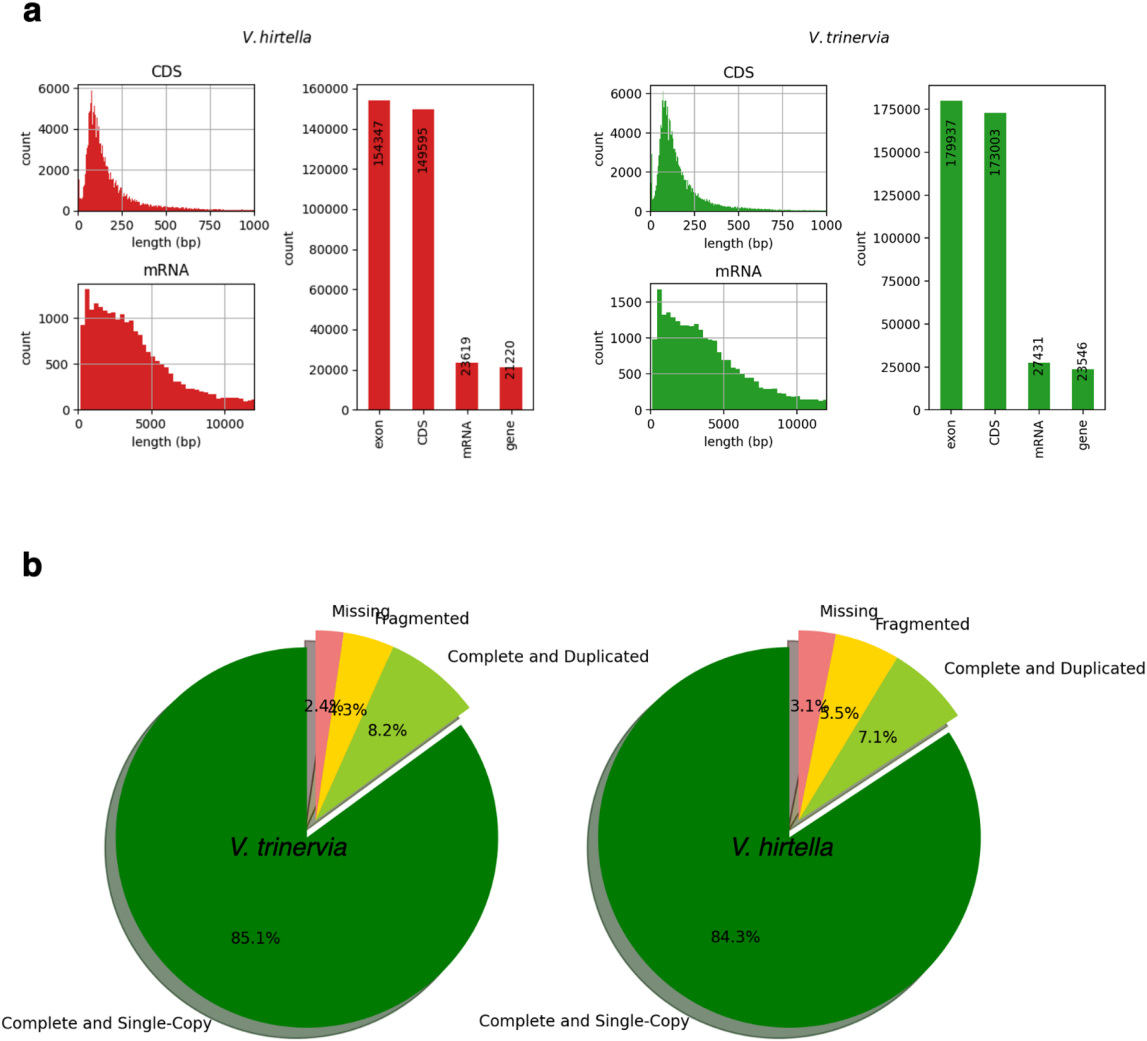
Comparative analysis of the gene prediction profiles of *V. hirtella*. and *V. trinervia* (**a**) A comprehensive visualization of the predicted gene catalogs of the *V. hirtella* and *V. trinervia* genomes, allowing a detailed exploration of their genomic landscapes. (**b**) Evaluations of the quality and completeness of the genome assemblies, using the Benchmarking Universal Single-Copy Orthologs (BUSCO) framework as a reliable metric for assessment.

Both *V. hirtella* and *V. trinervia* were subjected to de novo assembly using Illumina sequencing, incorporating paired-end and mate-pair library preparation methods. The resulting assemblies showed satisfactory contiguity, as indicated by N50 values of 209.5 Kb and 496.6 Kb, respectively. Repeat profiling of the assembled genomes revealed high similarity in their repetitive element profiles, with retroelements accounting for approximately 10 to 11 percent of the total genome sequence (Supplementary Table S2). The gene catalogs of *V. hirtella* and *V. trinervia* were assembled using a combination of ab initio and homology-based methods, complemented by transcriptomic data. A comparison of gene abundance in each species revealed 21,220 genes in *V. hirtella* and 23,546 genes in *V. trinervia*. The observed distribution patterns of mRNA length and coding sequence length (CDS) were comparable for both species, providing a robust basis for subsequent investigations (Fig. 4a). Benchmarking of Universal Single-Copy Orthologs (BUSCO) revealed that more than 90% of the genes were complete in both species (Fig. 4b).

### Comparative analysis of *V. hirtella*, *V. trinervia,* and *V. reflexo-pilosa*

#### Gene family evolution of the three *Vigna* species

**Figure 5:**
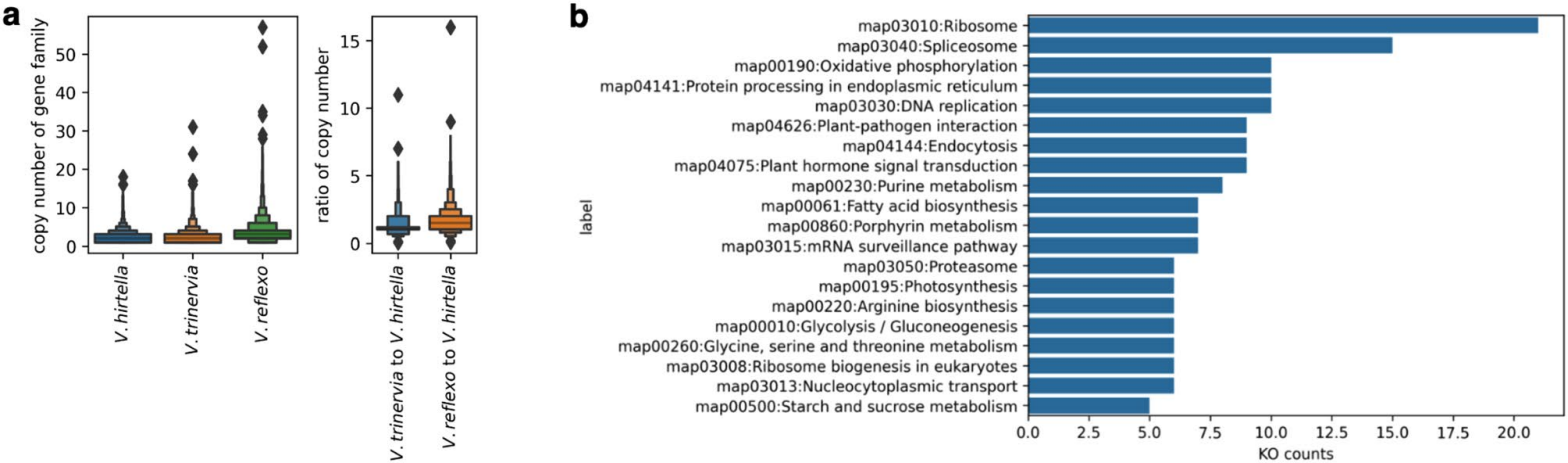
Copy number amplification in *V. reflexo-pilosa*. (**a**) Distribution of gene family copy numbers and copy number ratios among *V. trinervia*, *V. hirtella*, and *V. reflexo-pilosa*. (**b**) KEGG pathway representation of the gene family showing a twofold increase in *V. reflexo-pilosa* compared to *V. hirtella* and *V. trinervia*.

To elucidate the allopolyploid evolution of *Vigna* species, we performed comparative genomic analyses between our sequenced assemblies of *V. trinervia*, *V. hirtella* and the previously assembled *V. reflexo-pilosa* from our previous research^21^. Using the eggNOG database, we annotated the predicted gene catalog encompassing the three *Vigna* species and assigned corresponding eggNOG IDs indicating the respective gene families. Comparative analysis of copy numbers within each gene family revealed that *V. reflexo-pilosa* had a higher copy number distribution compared to the other *Vigna* species (Fig. 5a). Gene families within *V. reflexo-pilosa* that exhibited a twofold increase in copy number compared to the other *Vigna* species may represent the residual signatures of a polyploidization event. Study of these amplified gene families is essential to unravel the genomic implications of such polyploidization, including exploration of the affected biochemical pathways and elucidation of their functional consequences. Our analysis revealed 1221 gene families that fit this scenario. Further annotation of these gene families using the Kyoto Encyclopedia of Genes and Genomes (KEGG) database revealed specific pathways that were preferentially conserved after polyploidization^25^ (Fig. 5b). Notably, the "ribosome" and "spliceosome" pathways had twice as many copies in *V. reflexo-pilosa*. The observed trend of amplified ribosomal DNA (rDNA) is consistent with previous research^26^.

#### Comparative synteny analysis among three *Vigna* species and species tree construction

**Figure 6:**
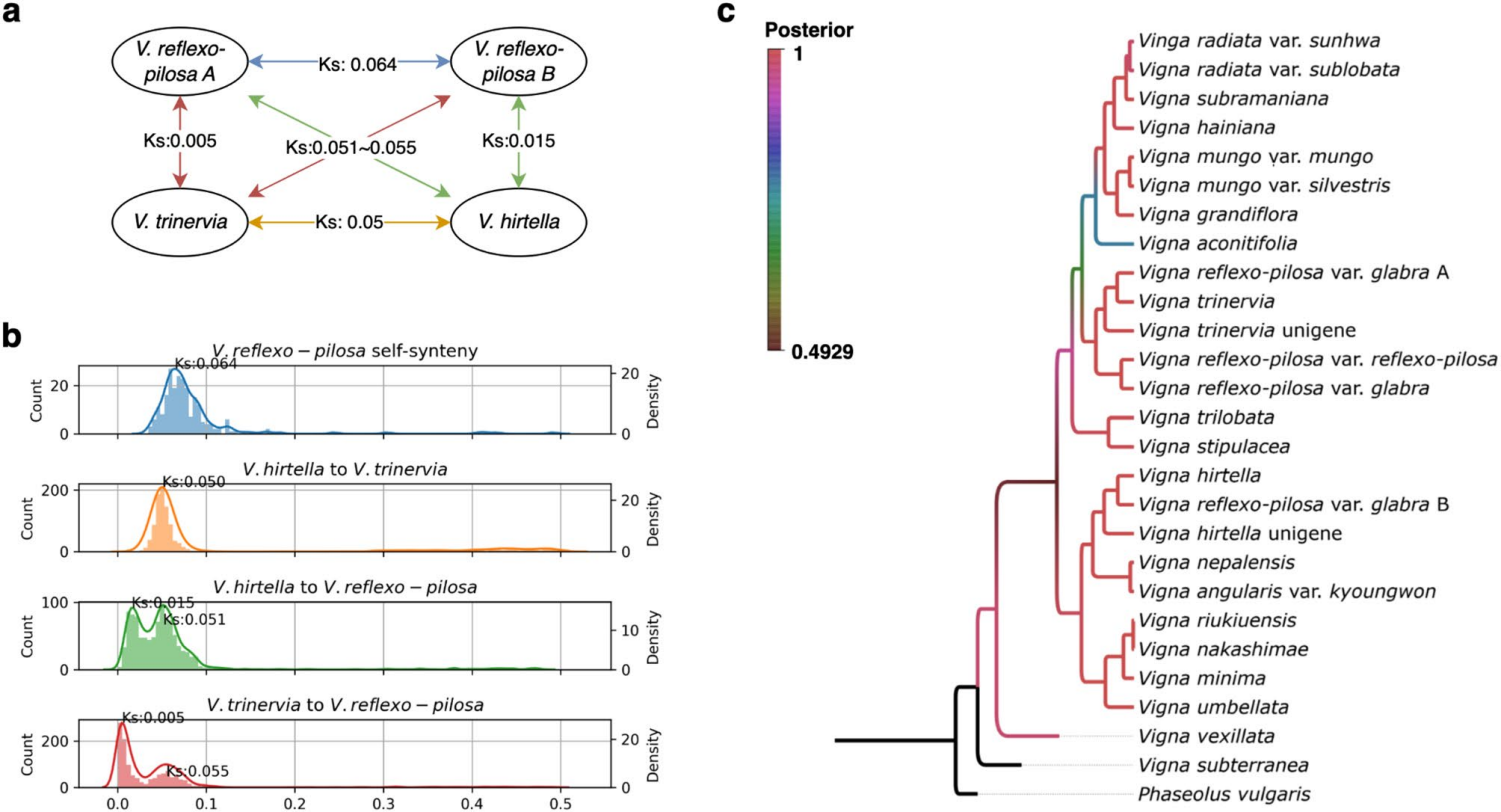
Demonstration of polyploidization of *V. reflexo-pilosa* derived from *V. hirtella* and *V. trinervia.* (**a**) A diagram showing the process of polyploidization of *V. reflexo-pilosa*, supported by Ks values. (**b**) Distribution of Ks values showing self-genome synteny in *V. reflexo-pilosa*, as well as synteny between *V. hirtella* and *V. reflexo-pilosa*, and *V. trinervia* and *V. reflexo-pilosa*. (**c**) A Bayesian species tree derived from genome and transcriptome data of different *Vigna* species, with the genome of *V. reflexo-pilosa* divided into A and B sub-genomes based on self-synteny analysis.

To validate the previously proposed donor species of *V. hirtella* and *V. trinervia*, we split the *V. reflexo-pilosa* genome into constituent A and B genomes (Fig. 6a). Self-synteny analysis of the *V. reflexo-pilosa* genome revealed synteny blocks that may indicate a past polyploidization event, with a modal synonymous substitution rate (Ks) of 0.064. Interestingly, synteny analysis between *V. hirtella* and *V. trinervia* revealed a comparable modal Ks value of 0.05, which is close to the self-synteny Ks value obtained for *V. reflexo-pilosa*. Comparative analysis of genetic divergence between *V. trinervia* and *V. reflexo-pilosa*, and between *V. hirtella* and *V. reflexo-pilosa*, yielded initial peak values of 0.015 and 0.005, respectively (Fig. 6b). These results suggest a possible genetic relationship: *V. trinervia* is closely aligned to the 'A' genome of *V. reflexo-pilosa*, while *V. hirtella* shows a greater affinity to the 'B' genome of *V. reflexo-pilosa* (Supplementary Figure S2). This finding provides compelling evidence to support the hypothesis that *V. hirtella* may be a plausible candidate as a donor species for *V. reflexo-pilosa*. Furthermore, using the orthologous genes identified through the bioinformatics pipeline, we constructed a phylogenetic tree using a Bayesian inference approach with the BEAST software^27^, which strongly suggests that *V. hirtella* is the most likely candidate for the donor genome, showing a closer genetic proximity to the B genome of *V. reflexo-pilosa* compared to other species within the genus *Vigna* (Fig. 6c).

**Figure 7:**
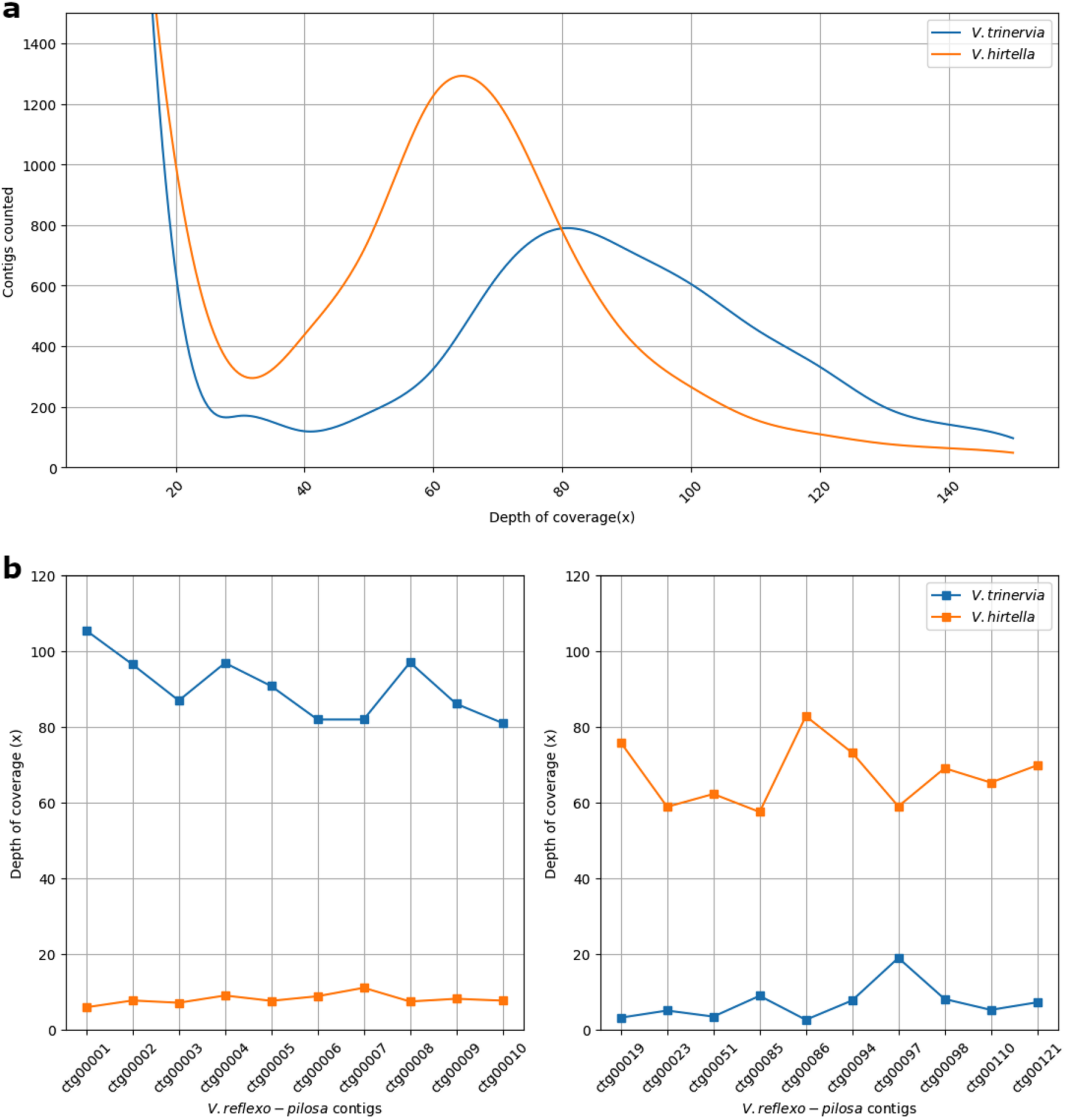
Paired-end reads from *V. hirtella* and *V. trinervia* were mapped to the *V. reflexo-pilos* genome. (**a**) The number of contigs observed varies with coverage, with a common trend of increased contig number occurring at depths below ×16. (**b**) Top10 contigs, ranked by length where *V. hirtella* and *V. trinervia* appear to be dominant at coverage depth.

When *V. hirtella* and *V. trinervia* sequences were mapped to the *V. reflexo-pilosa* genome, it was observed that despite average coverage depths of 39.4× and 50.2×, respectively, the contig-wise peak frequency ranges were 0–16 and 46–75 for *V. hirtella* and 0–16 and 55–106 for *V. trinervia* (Fig. 7a). Furthermore, it was found that *V. hirtella* and *V. trinervia* sequences aligned to each contig in a complementary manner (Fig. 7b, Supplementary Figure S2, and Supplementary Figure S3), and the total size of the distinct *V. hirtella*-dominant and *V. trinervia*-dominant contigs was approximately 309.5 Mb and 332.0 Mb, respectively (Table 1). Based on these results, it was possible to determine the ancestor from which the assembly-generated contigs of *V. reflexo-pilosa* is originated and contigs derived from *V. hirtella* appeared to be less capable of forming long connections compared to those from *V. trinervia*.

**Table 1:** Depth of coverage (DOC) was calculated for contigs corresponding to the N90 value in the *V. reflexo-pilosa* assembly results using paired-end data from *V. hirtella* and *V. trinervia*.

Mapped condition over contigs	Contig number	Total size	Average depth (standard deviation) of *V. trinervia*	Average depth (standard deviation) of *V. hirtella*
DOC under 16 × for *V. trinervia* reads	5284	309,549,858		70.4 (40.8)
DOC under 16 × for *V. hirtella* reads	4140	331,975,605	91.8 (35.7)	
DOC under 16 × for both species’ reads	11	300,539		

## Discussion

A phylogeny constructed from consensus sequences of chloroplast genomes is congruent with the established species phylogeny, despite the limitations of relying on the sequence used as a reference, which may not fully account for genome-wide structural variants. However, the uniparental inheritance of chloroplasts, predominantly from the maternal lineage, is a limitation in estimating all ancestors involved in hybridization or polyploidization events. In this study, we could only confirm one of the genome donors of *V. reflexo-pilosa* as *V. trinervia* or its closely related species. In our previous study^21^, it was proposed that the B genome donor of *V. reflexo-pilosa* belongs to the *Angulares* section of Asian *Vigna*; however, it is expected to exhibit a distant diversity point compared to other *Angulares* species. Given that, in our phylogenetic study using the consensus sequences of chloroplast genome, *V. hirtella* fulfills the criteria, it can be considered as another plausible candidate for the genome donor of *V. reflexo-pilosa*. Since both *V. hirtella* and *V. trinervia* are found in the Southeast Asian region, natural hybridization between these species, with *V. trinervia* as the maternal ancestor and *V. hirtella* as the paternal donor, seems plausible.

The de novo genome assembly and annotation of *V. hirtella* and *V. trinervia* using Illumina sequencing, along with RepeatMasker and our in-house analysis pipeline, provided insights into the genetic composition of these species. The similarity in repeat profiles, gene counts, mRNA length, and coding sequence length confirmed the reliability of the sequencing and assembly processes.

The observed higher gene family copy number in *V. reflexo-pilosa* suggests the presence of residual signatures from a previous polyploidization event. The enrichment of specific pathways in *V. reflexo-pilosa*, such as "ribosome" and "spliceosome" processes, provides insight into the impact of polyploidization on genome functionalization. The increased rDNA copy number in polyploid *Vigna* species may confer functional advantages that allow them to maintain higher levels of ribosome biosynthesis under stress conditions, potentially increasing their resilience to adverse environments^28^.

The synteny analysis, together with Ks value calculations, provides evidence that *V. hirtella* is a close relative and likely donor species to *V. reflexo-pilosa*. This finding is consistent with previous studies using genotype patterns derived from simple sequence repeat (SSR) markers^22^. The phylogenetic tree based on Bayesian inference further supports this hypothesis. However, we acknowledge that inferences based on genetic correlation can be affected by factors such as selective pressure, mutation rates, and genetic drift^29^. Therefore, further investigation with more accessions of *Vigna* species is warranted to gain a deeper understanding of speciation within this genus.

In general, when mapping self-sequences or sequences from related species to the target genome, variations in depth coverage can occur from contig to contig due to factors such as sequencing bias. However, in most cases, although there may be a bias toward lower depths, it tends to follow a Gaussian distribution centered around the average depth of the genome. When ancestral sequences were aligned directly to allopolyploidy, it was observed that the mapping pattern at the contig level was concentrated at much lower depths or slightly higher values than the average depth. These results can be attributed to the fact that sequences originating from each progenitor did not align well with sequences from other progenitors. This suggests that at the genome level of *V. reflexo-pilosa*, the sequences derived from each progenitor are well conserved and maintained in their respective forms. To validate this assumption, a comparison was made using the results of self-synteny analysis in 226 paired regions. The result of this analysis showed that, except for a single contig (Vreflexopilosa_ctg411) where a synteny block was detected within the contig itself, all other regions showed a bias towards higher depths in either *V. trinervia* or *V. hirtella*, supporting the notion that sequences from each ancestor tend to maintain their distinct characteristics within *V. reflexo-pilosa*.

Despite the similarities in genome size and gene prediction results between *V. hirtella* and *V. trinervia* during the assembly analysis using the same method, it was observed that fundamental assembly statistics such as the number of contigs and N50 values, exhibited less favorable results in *V. hirtella* than in *V. trinervia*. When examining the mapping of reads from each progenitor to *V. reflexo-pilosa*'s contigs, it was noted that longer contigs tended to have a higher depth of alignment with *V. trinervia* sequences. Connecting these observations, it implies that there might be specific factor hindering *V. hirtella* from making a substantial contribution to the assembly of longer contigs when utilizing short reads.

Future research should focus on understanding the influence of conserved and expanded gene families and the potential adaptive advantages conferred by increased rDNA copy numbers on the functional dynamics and adaptability of *V. reflexo-pilosa*. The observed rapid and dynamic evolution of the rDNA gene family, similar to previous studies in yeast^30^, may play an important role in enhancing the adaptability and domestication processes of plant species^31^.

In summary, our research provides genomic evidence for polyploidization within the genus *Vigna* and identifies potential donor species for allotetraploid species through de novo genome assembly. These findings provide valuable insights into the gene clusters affected by polyploidization, which may have important implications for plant adaptability and domestication processes. Thus, our research significantly advances the current understanding of plant evolution and the underlying mechanisms of plant adaptation.

## Methods

### Plant materials

*V. hirtella* was newly included in 22 accessions from 18 different *Vigna* species, including both Asian and African domesticated varieties that were mentioned in our previous study^21^. These accessions were collected from various national and international genebanks, namely Chai Nat Field Crops Research Center in Thailand, National Agrobiodiversity Center in Korea, National Institute of Agrobiological Sciences in Japan, National Botanic Garden of Belgium, Australian Collections of Plant Genetic Resources, International Center for Tropical Agriculture in Colombia, International Livestock Research Institute in Kenya, and International Institute of Tropical Agriculture in Nigeria.

### Consensus sequence of the chloroplast genome

RNA-seq data for each *Vigna* species sample were aligned to the chloroplast sequence (NC_013843.1) using BWA mem 0.7.17-r1188^32^. Duplicate reads were removed using sambamba v.0.6.8^33^ and variant calling was performed using SAMtools 1.9. Variants with a phred score of 30 or higher were used to generate a consensus sequence for *V. radiata* chloroplast DNA using bcftools 1.9. The consensus sequences of each accession were aligned using MAFFT v7.453^34^, and a neighbor-joining method with 1000 bootstrap replications was used for phylogenetic analysis on the resulting alignment.

We also performed gene prediction and annotation on the *Vigna* chloroplast consensus sequence using GeSeq 2.03^35^. Subsequently, we filtered regions representing comprehensive coverage by RNA-seq data and calculated nucleotide diversity (Pi) using DnaSP v6.12.03^36^. ETE3 v3.1.3^37^ was used to compare phylogenetic trees from consensus sequences and filtered regions.

Based on the constructed phylogenetic tree, we selected specific species and performed de novo assembly of chloroplast genomes using DNA sequences through GetOrganelle 1.7.7.9^38^ with default option.

### De novo assembly and annotation

To perform de novo assembly, we first estimated the genome size of the sample using Jellyfish v1.1.11^39^ with k-mer analysis at 17, 21, and 25 (Supplementary Figure S4). Platanus-allee v2.2.0^40^ software was used to perform de novo assembly with paired-end reads and mate pair reads of different insert sizes (350 bp for paired-end reads, 5 kb, and 10 kb for mate pair reads). Scaffolding and gap filling were performed on mate pair reads using SSPACE v2.1.1^41^, and the best scaffold was selected based on number of scaffolds, scaffold sum, and N50. A length cutoff was applied to remove short scaffolds (Supplementary Figure S5). The assembly results of the *V. reflexo-pilosa* genome were examined by directly aligning the paired-end reads of *V. hirtella* and *V. trinervia* to assess the overall mapping pattern.

Repeat masking was performed using the RepeatModeler 2.0.4^42^ and RepeatMasker 4.1.5^43^ pipelines to identify and mask repetitive elements in the assembled genome. RepeatModeler was used to generate a de novo repeat library, which was then used by RepeatMasker to mask repetitive elements in the genome sequence.

For genome-guided transcriptome assembly, RNA reads were mapped to the assembled DNA sequence using Tophat v2.0.13 software^44^, and the assembled transcriptome sequence was obtained from the resulting BAM file using Trinity r20140717^45^. Annotation of the assembled DNA sequence and transcriptome sequence data was performed using the Seqping v0.1.33 pipeline^46^, which included gene prediction models built using GlimmerHMM v3.0.4, AUGUSTUS v3.2.2, and SNAP 20120517 software^47,48^. Prediction results were combined with the MAKER v3.01.03^49^ annotation program included in the Seqping pipeline. Additional annotation was performed by searching consensus sequences against several databases, including UniProt^50^, GO^51,52^, InterPro^53^, Pfam^54^, TIGRFAM^55^, and eggNOG^56^ using blast v2.6.0 + software^57^.

### Comparative genomics

A multi-step process was used to identify true orthologs (Supplementary Figure S6). Synteny analysis using MCScanX^58^ was performed on the *V. reflexo-pilosa*, *V. trinervia*, and *V. hirtella* genomes to explore syntenic relationships within the reference genomes. Self-synteny analysis was performed on *V. reflexo-pilosa* to detect matching regions, which were then partitioned based on the Ks value. The portion closer to *V. trinervia* was designated the ‘A’ genome, while the more distant portion was designated the ‘B’ genome.

Next, BLAST analysis was then performed to assign proteins from the *V. hirtella* and *V. trinervia* genome assemblies to each transcriptome assembly of the 22 *Vigna* accessions from the previous study^21^ to identify candidate orthologs. Gene family relationships between the transcriptome assemblies and the assembled genomes were determined using the eggNOG database^56^. Proteins identified as matches in both the BLAST result and the eggNOG database search were classified as true orthologs.

### Supplementary Information

Supplementary Information.

## Data Availability

Raw sequence reads are deposited in the SRA (BioProject: PRJNA961890). The assembled sequences of *V. reflexo-pilosa, V. trinervia* and *V. hirtella* are available on NCBI with BioSampleID: SAMN34371969, SAMN34371970 and SAMN34371971.
