# Supplementary material for: Unraveling the maternal and paternal origins of allotetraploid Vigna reflexo-pilosa
Source: Sci Rep. 2023 Dec 22;13:22951. doi: 10.1038/s41598-023-49908-2 (PMC10746702; doi:10.1038/s41598-023-49908-2)
Supplement: Supplementary file 1 — Supplementary Information. [file 41598_2023_49908_MOESM1_ESM.docx]

**Supplementary Figures**


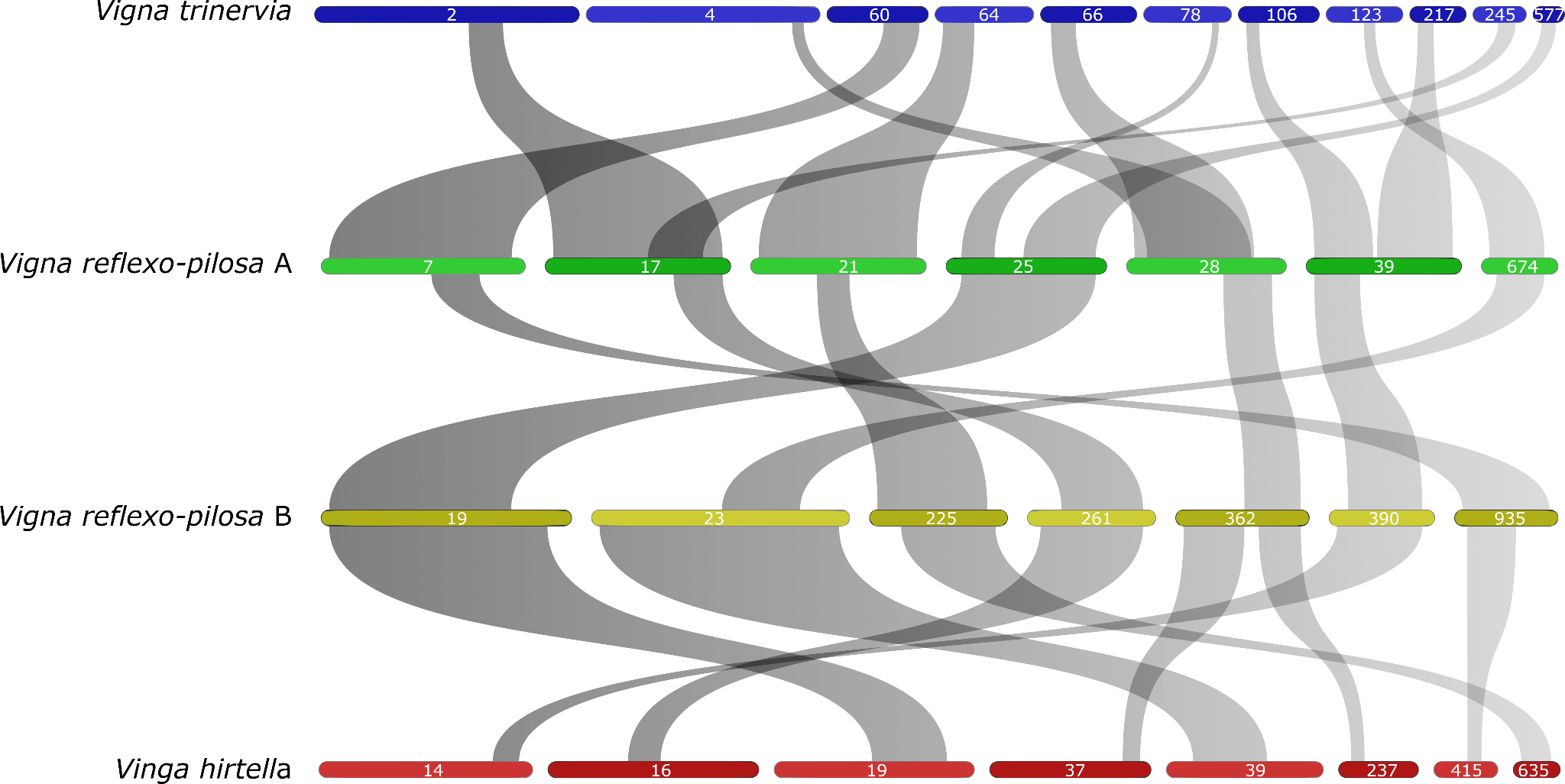


**Supplementary Figure S1.** Synteny analysis results to represent the relationship among *V. hirtella*,

*V. trinervia*, and *V. reflexo-pilsoa*. The contigs of *V. reflexo-pilosa* were divided into A and B genomes based on the Ks values from self-synteny analysis, indicating the linkage between *V. trinervia* and the A genome, as well as *V. hirtella* and the B genome.


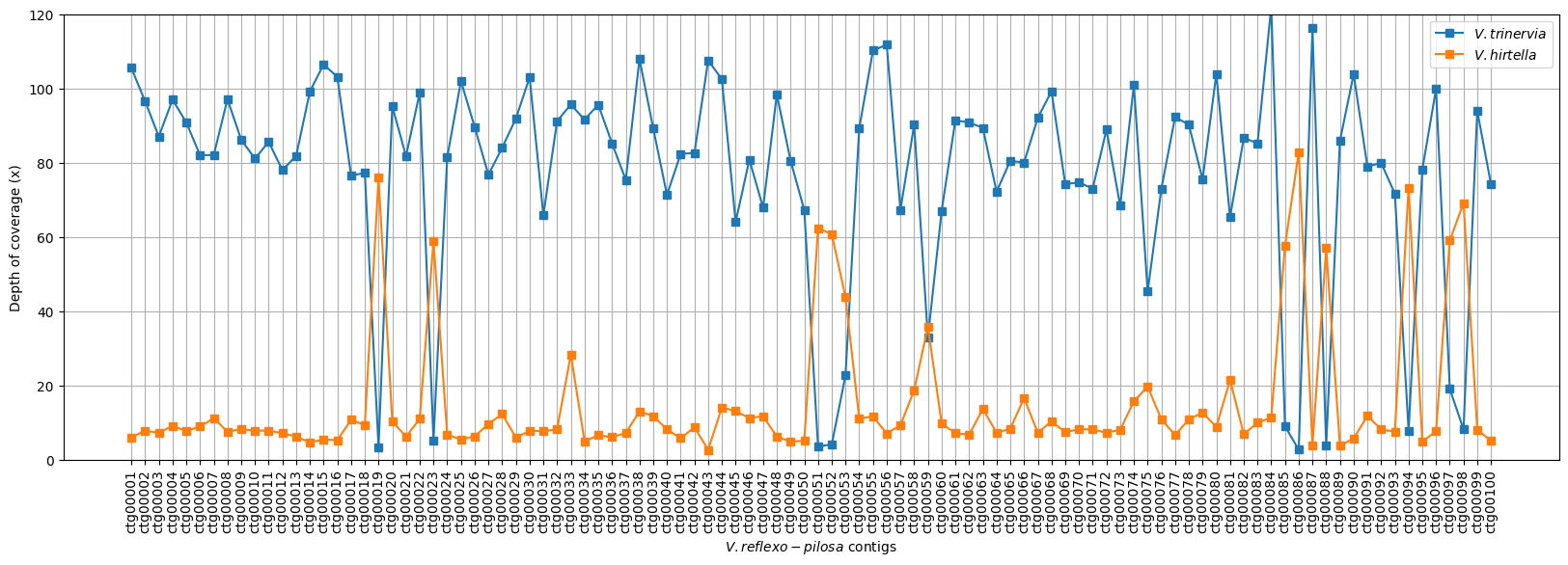


**Supplementary Figure S2.** Depth of coverage pattern of *V. trinervia* and *V. hirtella’s* paired-end reads aligned over the top 100 contigs, ranked by length, of the *V. reflexo-pilosa* genome.


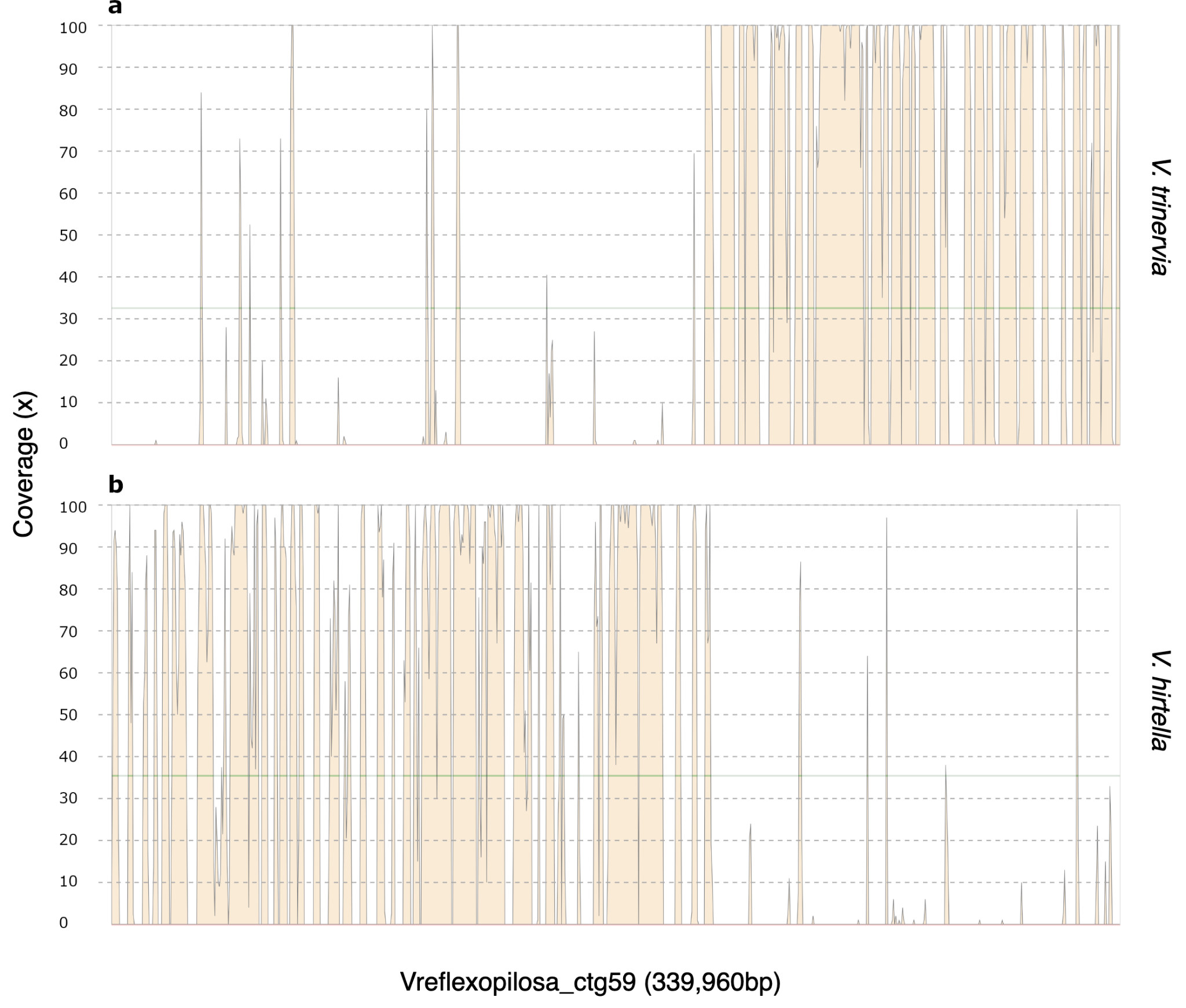


**Supplementary Figure S3.** When examined at the contig level, the alignment patterns of (a) *V. trinervia* and (b) *V. hirtella* reads were evident in contig Vreflexopilosa_ctg59, which showed similar depth of both progenitors. Although this contig appeared as a single unit, there was a clear distinction between progenitors in different regions, and a similar pattern was observed in other contigs as well.
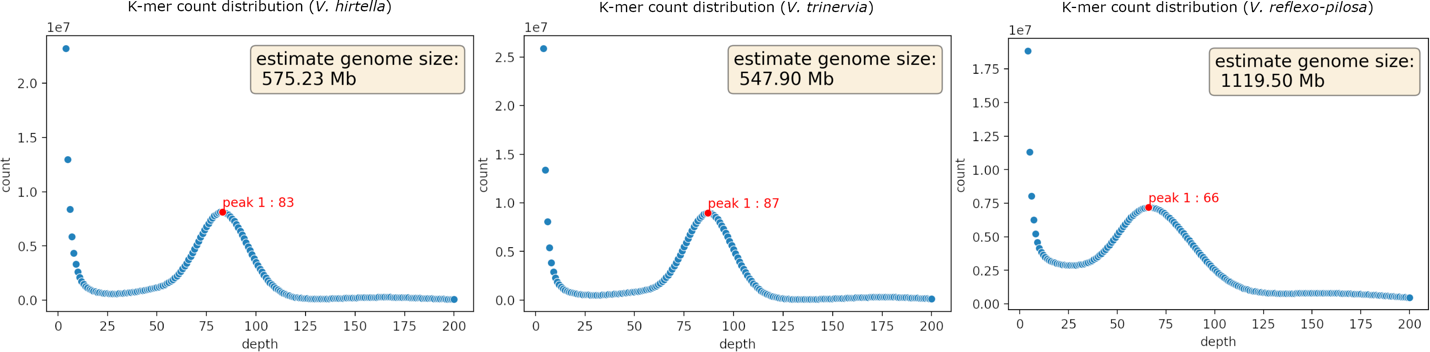


**Supplementary Figure S4.** A comparative analysis of genome sizes among three different plant species: *V. hirtella*, *V. trinervia*, and *V. reflexo-pilosa*. Genome size estimation was performed, employing k-mer length of 21.


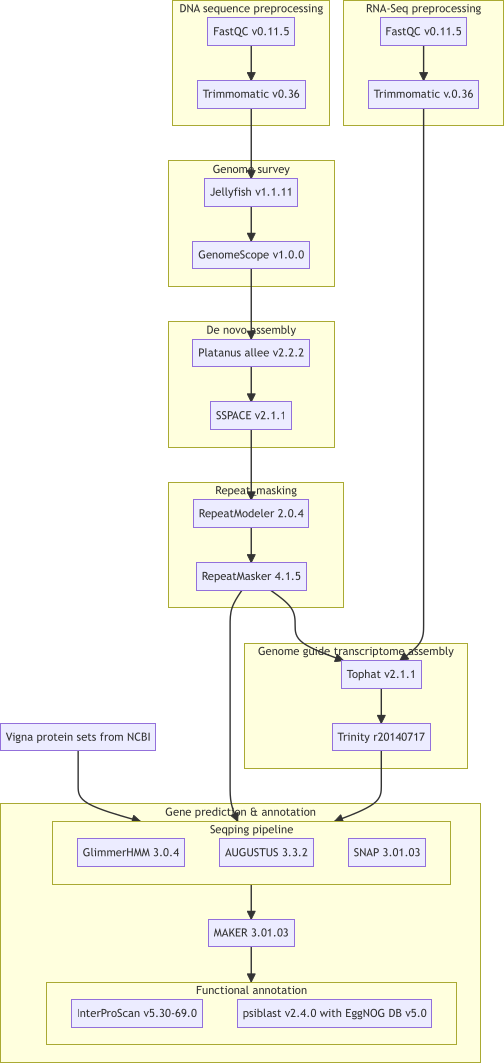


**Supplementary Figure S5.** Schematic representation of the bioinformatics workflow implemented for *de novo* whole genome assembly and subsequent analysis.


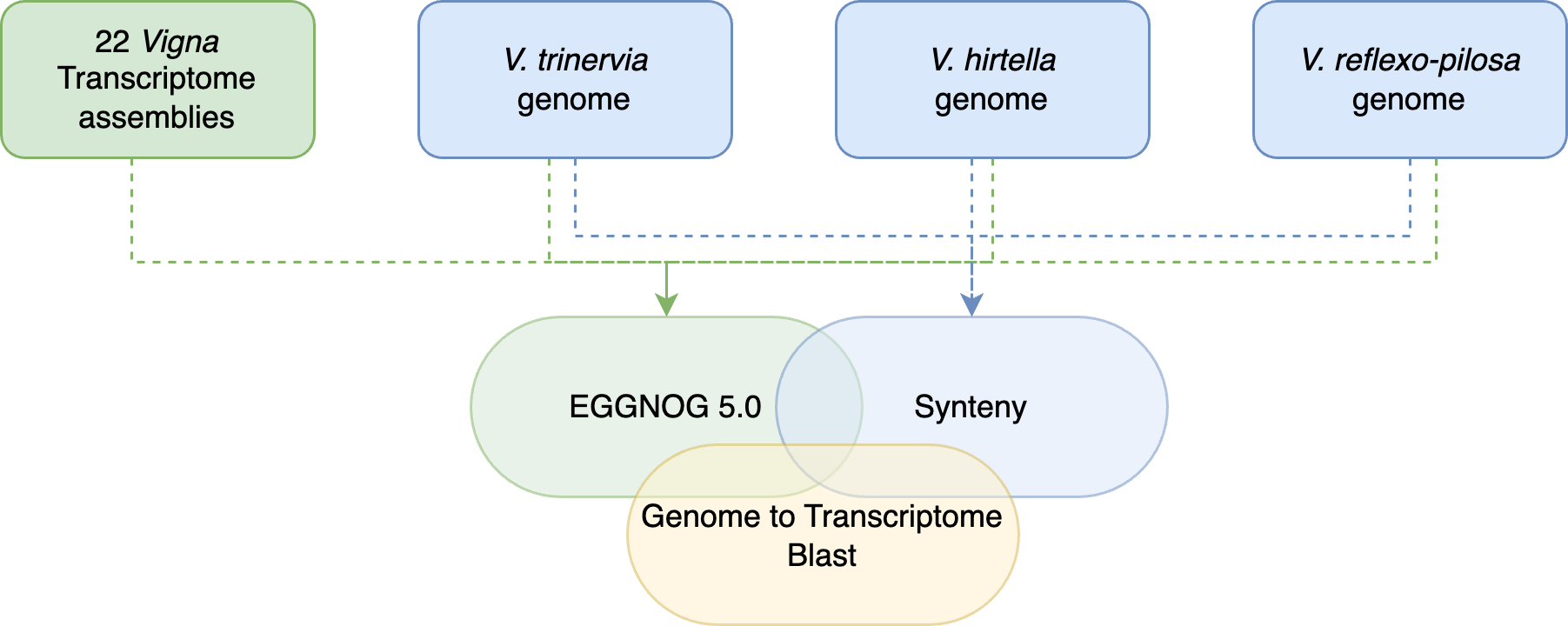


**Supplementary Figure S6. S**chematic representation of the methodology used to retrieve true orthologous genes from different *Vigna* species using both genome and transcriptome assembly data.

**Supplementary Tables**

|  | **BEST RANK COUNTED ON CHLOROPLAST GENOME *DE NOVO* ASSEMBLY RESULT OF** | | |
| --- | --- | --- | --- |
| **40 PREDICTED GENES FROM CONSENSUS SEQUENCE OF** | ***Vigna trinervia*** | ***Vigna hirtella*** | ***Vigna reflexo-pilosa*** |
| *Vigna aconitifolia* | 27 | 20 | 26 |
| *Vigna angularis* var. *kyoungwon* | 17 | 26 | 17 |
| *Vigna grandiflora* | 25 | 19 | 24 |
| *Vigna hainiana* | 22 | 18 | 21 |
| *Vigna hirtella* | 17 | 34 | 17 |
| *Vigna minima* | 19 | 23 | 19 |
| *Vigna mungo* var. *mungo* | 23 | 18 | 22 |
| *Vigna mungo* var. *silvestris* | 23 | 18 | 22 |
| *Vigna nakashimae* | 20 | 25 | 20 |
| *Vigna nepalensis* | 20 | 31 | 20 |
| *Vigna radiata* var. *sublobata* | 22 | 18 | 21 |
| *Vigna radiata* var. *sunhwa* | 23 | 19 | 22 |
| *Vigna reflexo-pilosa* var. *glabra* | 34 | 18 | 37 |
| *Vigna reflexo-pilosa* var. *reflexo-pilosa* | 34 | 18 | 37 |
| *Vigna riukiuensis* | 18 | 24 | 18 |
| *Vigna stipulacea* | 21 | 16 | 20 |
| *Vigna subramaniana* | 22 | 18 | 21 |
| *Vigna subterranea* | 11 | 10 | 11 |
| *Vigna trilobata* | 23 | 19 | 22 |
| *Vigna trinervia* | 38 | 19 | 35 |
| *Vigna umbellata* acc.2004T2 | 19 | 22 | 19 |
| *Vigna umbellata* acc.CIAT34386 | 17 | 21 | 17 |
| *Vigna vexillata* | 13 | 11 | 14 |

**Supplementary Table S1.** BLAST analysis of 40 predicted gene sequences derived from 23 accessions used for Pi calculation against the chloroplast *de novo* assembly results of *V. trinervia*, *V. hirtella*, and *V. reflexo-pilosa*. Ranking was based on pident, bit-score, and HSP in that order.

| **LIBRARY** | **ACCESSION** | **SPECIES** | **TOTAL LENGTH** | **READS NUM** | **GC RATIO** | **Q20** | **Q30** |
| --- | --- | --- | --- | --- | --- | --- | --- |
| Vhirtella_PE | W8-3 | *Vigna hirtella* | 59,679,843,854 | 395,230,754 | 36.42 | 93.64 | 86.79 |
| Vhirtella_10kb | W8-3 | *Vigna hirtella* | 42,683,851,312 | 282,674,512 | 39.47 | 92.53 | 85.69 |
| Vhirtella_5kb | W8-3 | *Vigna hirtella* | 68,300,754,276 | 452,322,876 | 39.1 | 89.92 | 81.14 |
| Vreflexo_PE | V1160 | *Vigna reflexo-pilosa* var. *glabra* | 92,358,773,704 | 914,443,304 | 37.41 | 96.28 | 91.45 |
| Vreflexo_5kb | V1160 | *Vigna reflexo-pilosa* var. *glabra* | 25,466,802,358 | 252,146,558 | 36.23 | 97.98 | 93.96 |
| Vtrinervia_PE | AusTRCF319618 | *Vigna trinervia* | 59,584,186,260 | 394,597,260 | 35.58 | 92.15 | 83.67 |
| Vtrinervia_10kb | AusTRCF319618 | *Vigna trinervia* | 68,582,751,004 | 454,190,404 | 37.06 | 90.74 | 82.61 |
| Vtrinervia_5kb | AusTRCF319618 | *Vigna trinervia* | 55,381,400,844 | 366,764,244 | 40.79 | 90.83 | 84.08 |

**Supplementary Table S2.** Statistical analysis of raw sequencing data for *de novo* genome assembly.

| **SPECIES** | **CONTIG NUM** | **TOTAL SIZE** | **N50** | **MAX** | **MIN** | **AVG** |
| --- | --- | --- | --- | --- | --- | --- |
| *Vigna hirtella* | 9,957 | 468,319,003 | 233,709 | 2,053,433 | 1,000 | 47,034 |
| *Vigna trinervia* | 5,286 | 507,235,815 | 569,474 | 3,731,912 | 1,000 | 95,958 |
| *Vigna reflexo-pilosa* var. *glabra* | 28,571 | 794,358,682 | 84,532 | 758,200 | 1,000 | 27,802 |

**Supplementary Table S3.** Comprehensive statistics for *de novo* genome assembly using Platanus-allee v2.20 for assembly operations and SSPace for scaffolding procedures.
